# Screening and Bioinformatics Analysis of Crucial Gene of Heart Failure and Atrial Fibrillation Based on GEO Database

**DOI:** 10.3390/medicina58101319

**Published:** 2022-09-21

**Authors:** Yuansong Zhuang, Zhentao Qiao, Xuanye Bi, Dongjian Han, Qingjiao Jiang, Yi Zhang, Fuhang Wang, Miaomiao Liu, Quanxu An, Jiahong Shangguan, Deliang Shen

**Affiliations:** 1 Cardiology Department, First Affiliated Hospital of Zhengzhou University, Zhengzhou 450052, China; 2 Department of Vascular and Endovascular Surgery, First Affiliated Hospital of Zhengzhou University, Zhengzhou 450052, China

**Keywords:** heart failure, atrial fibrillation, GEO, bioinformatics analysis

## Abstract

*Background and objectives:* In clinical practice, we observed that the prognoses of patients with heart failure and atrial fibrillation were worse than those of patients with only heart failure or atrial fibrillation. The study aims to get a better understanding of the common pathogenesis of the two diseases and find new therapeutic targets. *Materials and Methods:* We downloaded heart failure datasets and atrial fibrillation datasets from the gene expression omnibus database. The common DEGs (differentially expressed genes) in heart failure and atrial fibrillation were identified by a series of bioinformatics methods. To better understand the functions and possible pathways of DEGs, we performed Gene Ontology (GO) and Kyoto Encyclopedia of Genes and Genomes (KEGG) analyses. *Results:* We identified 22 up-regulated genes and 14 down-regulated genes in two datasets of heart failure and 475 up-regulated and 110 down-regulated genes in atrial fibrillation datasets. In addition, two co-upregulated (FRZB, SFRP4) and three co-downregulated genes (ENTPPL, AQP4, C1orf105) were identified. GO enrichment results showed that these common differentially expressed genes were mainly concentrated in the signal regulation of the Wnt pathway. *Conclusions:* We found five crucial genes in heart failure and atrial fibrillation, which may be potential therapeutic targets for patients with heart failure and atrial fibrillation.

## 1. Introduction

Cardiovascular diseases (CVDs) represent the leading cause of death and disability in developed countries. Among them, heart failure is the final stage of various cardiac diseases that affects nearly 5 million people in the United States [1]. The one-year post-discharge mortality from chronic heart failure (CHF) is approximately 20–25%, which comes close to or surpasses many malignancies [2]. As a chronic and fatal condition, CHF accounts for more than 2 million hospitalizations per year in the United States, and the total annual costs are greater than USD 30 billion. Furthermore, multiple studies have observed that 10–50% of patients in terminal heart failure have comorbid atrial fibrillation (AF) [3,4,5,6]. Like heart failure, there is a significant risk of morbidity and mortality, as well as a decreased quality of life and an increase in healthcare costs associated with AF [7,8]. Most available evidence suggests that AF is a risk factor for death by CHF, independent of the presence of all traditional risk factors [3,4,5,6,9,10,11].

The underlying complex mechanisms between heart failure and atrial fibrillation pose a special challenge to daily clinical practice. Currently, catheter ablation and anti-arrhythmic drugs are the primary therapeutic approaches for restoring and maintaining sinus rhythm [12]. However, antiarrhythmic drugs used to treat patients with left ventricular dysfunction have a poor efficiency and present a high risk of adverse events. Moreover, interventional operations have many contraindications as well [13]. Based on the aforementioned, there is a need for the optimization of the current treatment plan for AF onset in heart failure patients. Consequently, it is crucial to fully understand the molecular mechanism and pathogenesis of HF and AF to improve the early diagnosis, treatment, and prognosis of these special patients.

Bioinformatic approaches based on microarray gene expression profiling provide novel opportunities to uncover the underlying mechanism of the two diseases at the molecular level [14]. This is useful as only limited data are reported about interactions among differentially expressed genes and key genes involved in the signaling pathways of HF and AF. We sought to leverage public sequence databases to identify common differentially expressed genes and key signaling pathways between HF and AF and to provide insights for the development of clinically therapeutic targets.

## 2. Materials and Methods

### 2.1. Data Source

We used “heart failure” and “atrial fibrillation” as key words to retrieve data sets from the National Center of Biotechnology Information (NCBI) GEO database (https://www.ncbi.nlm.nih.gov/geo/ (accessed on 20 March 2022)), and obtained two datasets (GSE120895, GSE57338) to describe heart failure caused by dilated cardiomyopathy and three datasets (GSE31821, GSE41177, GSE79768) to represent atrial fibrillation. The detailed information is illustrated in Table 1.

### 2.2. Data Preprocessing

Raw data in “CEL” format were downloaded from the gene expression omnibus (GEO) database and these datasets were subjected to background correction, normalization, and calculation of expression values using the robust multiarray average (RMA) normalization method implemented in the “Affy” library (version 1.72.0, Cambridge, Boston, Massachusetts, USA) [15]. Probes with missing expression values and genes of unknown function were removed, the probe names were converted to gene symbols using the platform annotation information, and the average expression values of multiple probes that corresponded to one gene were considered as the expression value of this gene. Three datasets from AF were merged by the same platform. The ComBat function from the sva package (3.42.0, Baltimore, MD, USA) in R was used to remove the batch effects of the datasets [16]. The final dataset was analyzed as the expression profile of atrial fibrillation. 

### 2.3. Identification of Differentially Expressed Genes

The raw microarray results were analyzed using the Limma package (version 3.50.1, Melbourne, Australia) of the R statistics suite. Differential expressed genes were selected with threshold of log_2_FC > 0.58 (log_2_1.5) or log_2_FC < −0.58 (−log_2_1.5) and *p* value < 0.05. Differentially expressed genes with log_2_FC > 0.58 were considered as up-regulated genes, and log_2_FC < −0.58 as down-regulated genes. All DEGs from HF and AF were run in the online Venn diagrams tool (http://bioinformatics.psb.ugent.be/webtools/Venn/ (accessed on 21 March 2022)) to identify the common DEGs between two diseases. Heatmap of DEGs was made by ggplot2 package (version 3.3.5, Houston, TX, USA) in R.

### 2.4. GO and KEGG Enrichment Analysis

KEGG (Kyoto Encyclopedia of Genes and Genomes) metabolic pathways and involved metabolites annotation were downloaded from KEGG API (https://www.kegg.jp/kegg/rest/keggapi.html (accessed on 21 March 2022)). GO (Gene Ontology) annotation of the target genes was performed using clusterProfiler (version 3.14.3, Guangzhou, China) and org.Hs.eg.db (version 3.14.0, Washington, DC, USA) packages in R. The minimum gene set was set to 5, the maximum set was 5000, and *p* value < 0.05 was considered statistically significant. The enrichment results were visualized using the R package ggplot2 (version 3.3.5, Houston, TX, USA).

## 3. Results

### 3.1. Common Up- and Down-Regulated Genes in HF and AF Datasets

**Figure 1:**
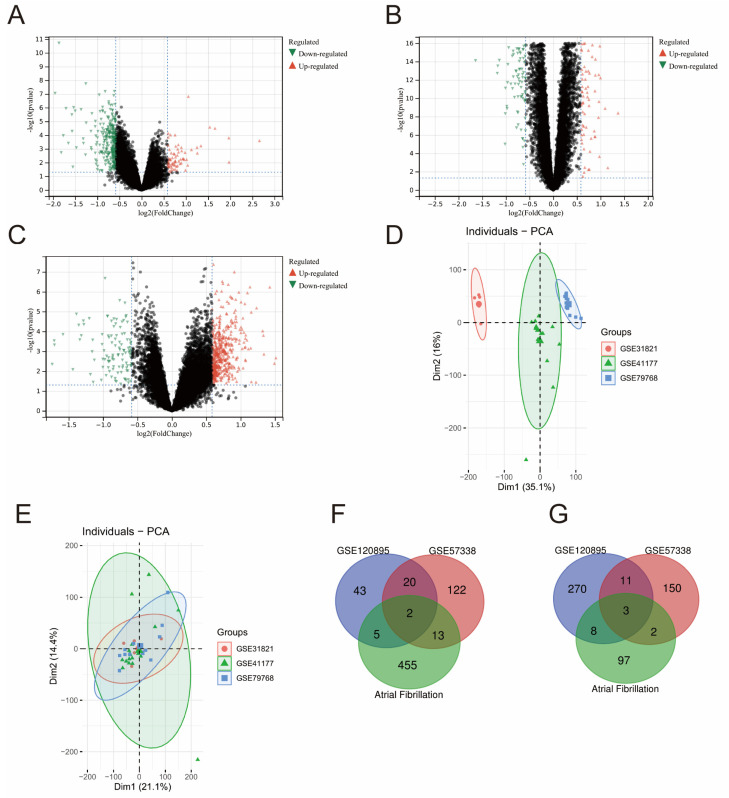
Volcano plot of gene expression, PCA analysis result, and Venn diagrams of differentially expressed genes in the two datasets. Panels (**A**–**C**) are volcano plots of gene expression in GSE120895, GSE57338, and the combined dataset of AF, respectively (red points represent up-regulated genes in dataset, green represent down-regulated genes in dataset). Panel (**D**) shows PCA clustering results of three datasets representing atrial fibrillation before datasets were combined. Panel (**E**) shows PCA clustering results of three datasets representing atrial fibrillation after datasets were combined. Panels (**F**,**G**) show the overlap of up-regulated and down-regulated genes in datasets.

The gene expression dataset GSE120895 was downloaded from the GEO database, and 362 HF-related DEGs were obtained by a differential analysis, of which 70 were highly expressed (*p* < 0.05, log2FC > log21.5) and 292 were poorly expressed ((*p* < 0.05, log2FC < −log21.5), Figure 1A). Similarly, from GSE57338 dataset of the patients with HF and the healthy subjects, a total of 323 genes were extracted, among which 157 genes were up-regulated and 166 genes were down-regulated (Figure 1B). Principle component analysis (PCA) plots showed the presence of distinct batches in the AF datasets (Figure 1D). To reduce the batch effects between these datasets, the merged dataset was normalized by applying the ComBat algorithm in R, which works by adjusting the data based on a known batch effect, and a PCA was performed (Figure 1E). The limma approach was subsequently used to analyze the differentially expressed genes in the merged datasets and 475 up-regulated and 110 down-regulated genes were identified. The results illustrated that there are significant differences in the expression of multiple genes in patients with HF and AF.

In order to visualize the results and further determine the common DEGs that exist in both datasets, Venn diagrams were plotted (Figure 1F,G). The result confirmed five common DEGs, which comprise two up-regulated genes and three down-regulated genes. To unveil the expression patterns of the DEGs among all subjects, we constructed a cluster heatmap to show the cross-correlation of DEGs among each individual, and the top 50 DEGs were selected based on the *p*-values (*p* < 0.05) (Figures S1–S3, Supplementary Material).

### 3.2. GO Enrichment Terms in HF and AF Datasets

**Figure 2:**
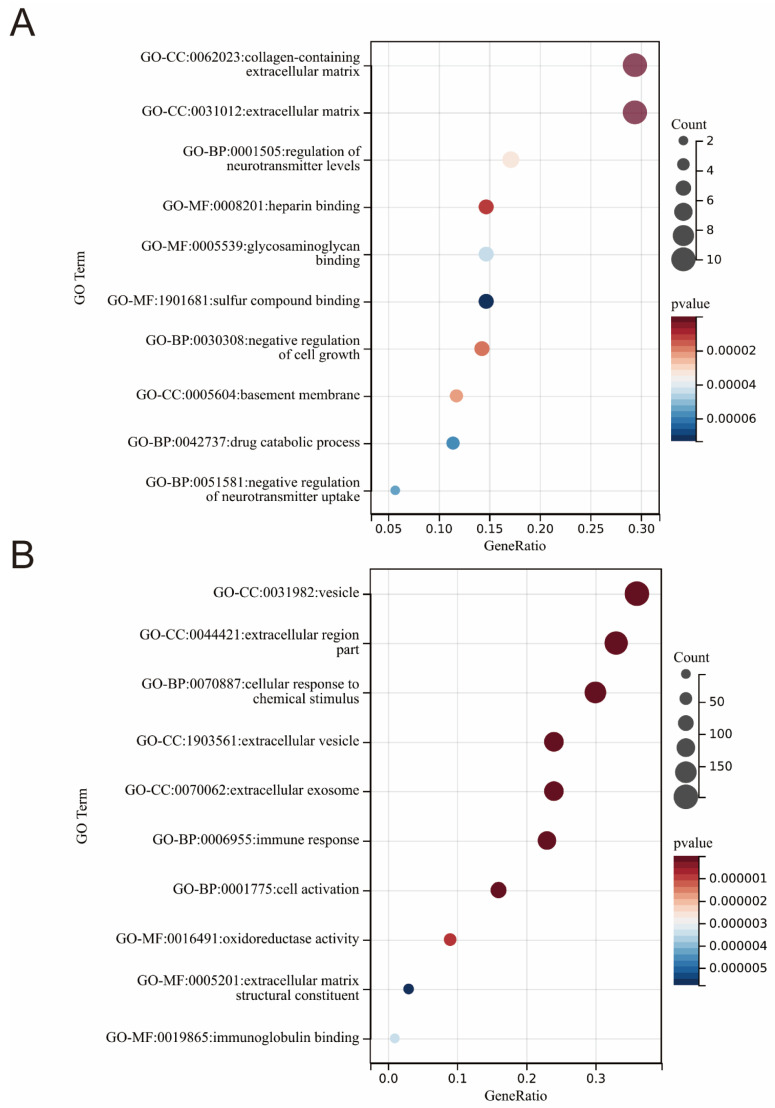
GO enrichment analysis of DEGs. Panel (**A**) shows the top 10 GO enrichment results in HF dataset. Panel (**B**) shows the top 10 GO enrichment results in AF dataset. In this figure, the X-axis represents the proportion of the current number of genes in GO terms, and the Y-axis represents the enriched GO terms (GO: Gene Ontology, BP: Biological Process, MF: Molecular Function, CC: Cell components), bubble colors from red to blue represent high to low importance, and bubbles from small to large represent counts of DEGs. DEGs: differentially expressed genes.

To gain insight into the cellular component (CC), molecular function (MF), and biological process (BP) of the DEGs products, we performed a gene ontology analysis. The GO analysis extracted from heart failure patients and healthy subjects revealed that DEGs were significantly enriched in the following biological processes (BP): the negative regulation of cell growth (GO:0030308), neurotransmitter content and the uptake regulation (GO:0001505, GO:0051581), and the drug catabolic process (GO:0042737). The cell component (CC) analysis revealed that DEGs were predominantly located in the extracellular matrix (GO:0062023) and basement membrane (GO:0005604). In the MF category, the DEGs were mainly enriched in heparin binding, glycosaminoglycan binding, and sulfur compound binding (GO:0008201, GO:0005539, GO:1901681) (Figure 2A).

Similarly, from the combined datasets of GSE31821, GSE41177, GSE79768 in patients with atrial fibrillation and healthy subjects, the BP terms of DEGs are mainly enriched in the cellular response to a chemical stimulus (GO:0070887), immune response (GO:0006955) and cell activation (GO:0001775). For the MF term, differentially expressed genes are mainly enriched in oxidoreductase activity (GO:0016491), immunoglobulin binding (GO:0019865), and in extracellular matrix structural constituent (GO:0005201); lastly, for the CC term, they were involved in the extracellular region (GO:00044421) and the extracellular vesicle (GO:1903561) (Figure 2B).

However, different terms from above results were found when performing the GO enrichment analysis of common DEGs of two diseases. It was found that for the BP term, the common DEGs were mostly enriched in the regulation of epithelial cell differentiation (GO:0090090) and the negative regulation of the canonical Wnt signaling pathway (GO:0030856), while for the MF term, they were mostly enriched in Wnt protein binding (GO:0017147) (Table 2). These results demonstrated that the Wnt signaling pathway is likely to contribute to the HF and AF.

### 3.3. KEGG Pathways Identified in HF and AF Datasets

Next, to explore the mechanism of disease occurrence and development, we used R software to identify the KEGG pathway of the DEGs from the two diseases. In the dataset representing heart failure, three pathways were determined: (1) the cGMP-PKG signaling pathway, (2) bile secretion, and (3) biosynthesis of amino acids (Table 3). Likewise, the top 10 pathways identified from the AF dataset include: (1) leishmaniasis, (2) staphylococcus aureus infection, (3) phagosome, (4) oxidative phosphorylation, (5) intestinal immune network for IgA production, (6) Parkinson’s disease, (7) Huntington’s disease, (8) viral myocarditis, (9) cell adhesion molecules (CAMs), and (10) Alzheimer’s disease (Table 4). However, significant KEGG pathways were not enriched in the common DEGs of the two diseases; one possible reason for this is that the number of common genes is too small (only five).

## 4. Discussion 

It is widely believed that HF and AF contain complex biological processes of multiple factors and stages. In recent years, a large number of novel biomarkers have been developed for early diagnosis, pathological mechanism research, and drug target screening in the two diseases [17]. However, our understanding of the pathogenesis of HF and AF at the genetic level remains extremely limited. In the present study, bioinformatic methods were used to analyze the microarray data of patients with heart failure and with atrial fibrillation, and we obtained the common DEGs and protein molecular networks of the two data sets of healthy subjects and patients with heart failure or atrial fibrillation. Finally, five genes (FRZB, SFRP4, ETNPPL, AQP4, C1orf105) were found to be highly co-expressed in patients with heart failure and atrial fibrillation. As far as we are aware, this is the first study to investigate the correlation between all differentially expressed genes between the heart failure and atrial fibrillation datasets. The findings may contribute to a better understanding of the mechanisms underlying the pathogenesis of HF and AF.

Considerable experimental and clinical data have shown that HF is a complicated pathophysiological process in which oxidative stress, calcium overload, myocardial remodeling, inflammatory activation, and myofibroblast activation serve important roles [18]. All of the above factors may participate in the atrial extracellular matrix and electrical remodeling, resulting in high AF inducibility [19]. This is analogous to a positive feedback mechanism that amplifies instability, further aiding in the complexity of the disease.

FRZB (frizzled-related protein, SFRP3) and SFRP4 are both members of the SFRP family. It is currently thought that SFRPs, as inhibitors of Wnt signaling, compete with the frizzled receptor for Wnt ligands based on their structural resemblance, and participate in physiological and pathological processes, such as embryonic development, tissue proliferation and differentiation, and anti-apoptosis [20,21]. Typically, the cardiovascular system of healthy adults depends on regulated canonical Wnt signaling precision, and numerous studies have shown that the abnormal activation of the Wnt/β-catenin signaling pathway is associated with cardiovascular diseases [22]. Askevold and Ren et al. suggested that the abnormal activation of FRZB in human heart failure and hypertension is a significant cause of disease progression [23,24]. Some researchers have reported that increased SFRP4 expression in the rat myocardial infarction model and in human coronary heart disease serum have also been observed and the knock-out of SFRP4 leads to significantly lower heart damage following an ischemia-reperfusion injury as compared to wild-type animals [25,26,27]. Moreover, our study found that despite the upregulation of the expression of FRZB in the heart tissue of patients with HF and AF, the gene expression of β-catenin downstream of the Wnt pathway was still upregulated. A similar phenomenon was also found in a previous study [28]. Wolke et al. [28] considered that the up-regulation of FRZB cuts off any potentially harmful signal factor input through the Wnt pathway. Mechanistically, the activation of β-catenin in AF may be due to increased Akt/GSK-3b/β-catenin signaling, a pathway that has only recently been shown to induce atrial fibrosis [29]. However, further studies are still needed in order to elucidate the roles of FRZB and SFRP4 in the growth of HF and AF.

ETNPPL (Ethanolamine-phosphate phospho-lyase), also known as AGXT2L1 (Alanine—glyoxylate aminotransferase 2-like 1), is located on chromosome 4q25. The gene product catalyzes the pyridoxal-phosphate-dependent breakdown of phosphoethanolamine, and converts it to ammonia, inorganic phosphate, and acetaldehyde. Some studies have shown that ETNPPL plays a certain role in glioma, gastric cancer, and liver fibrosis [30,31,32]. However, the specific function of ETNPPL remains unclear currently, especially in the study of cardiovascular disease. In our present study, ETNPPL as a significantly down-regulated gene in patients with dilated cardiomyopathy-induced heart failure and atrial fibrillation was detected. As a potential co-pathogenesis and therapeutic target of the two diseases, the role of ETNPPL in the cardiovascular system remains to be further studied.

AQP4 (aquaporin 4), a kind of membrane protein, is mainly distributed around the intercalated discs at the junction of cardiomyocytes. This protein mediates the permeability of water and small molecules across the cardiomyocyte membrane driven by an osmosis gradient, maintaining the water balance inside and outside the cell. Several studies have suggested that myocardial interstitial edema and endothelial damage can be observed in patients with heart failure and atrial fibrillation [33,34]. Additionally, Tan et al. found that the high expression of AQPs promotes myocardial inflammation and edema in doxorubicin-induced heart failure mice [35]. However, in the present study, we analyzed the data at the gene transcription level and found that AQP4 showed low expression significantly in the datasets of heart failure and atrial fibrillation. One possible reason for this discrepancy may be different causes of myocardial edema. Myocardial interstitial edema is mainly caused by fluid leakage in heart failure and atrial fibrillation, and the low expression of AQP4 aggravates the accumulation of fluid between myocardial cells. As a potential therapeutic target, the role of AQP4 in the pathogenesis of heart failure and atrial fibrillation deserves further attention.

C1orf105 is located on human chromosome 1q24, and its product-specific biological function is not clear. Zhang et al. [36] found that the transcription of C1orf105 was significantly lower in patients with atrial fibrillation than in those with sinus rhythm. A previous study reported that the C1orf105 gene was associated with the remodeling response to atherosclerosis [37]. In the present study, we found that C1orf105 showed a trend of low expression in both heart failure and atrial fibrillation patients, which indicated that C1orf105 was involved in the development of both diseases; it may become a new therapeutic target for patients with heart failure with atrial fibrillation.

However, the limitations of this study should not be ignored. Firstly, considering that the development of HF and AF results from various environmental and genetic factors, some unmeasured factors, such as family history and environmental risk factors of HF and AF, should be evaluated in further research. Moreover, five potential crucial genes need further validation by RT-qPCR in human heart tissue and the mechanisms on which these genes act are not completely clear. More evidence is required to find out the biological foundation. Finally, diastolic dysfunction was not included in this study, which is an important cause of heart failure. Further research should expand the sample size and target more diverse patients with heart failure with reduced and preserved ejection fraction.

## 5. Conclusions

In summary, five crucial candidate genes (FRZB, SFRP4, ETNPPL, AQP4, C1orf105) may play key roles in the occurrence and development of HF and AF, suggesting they may serve as potential biomarkers and therapeutic targets in the two diseases. 

## Figures and Tables

**Table 1:** Dataset baseline information.

GEO Serial Number	Diseases	Platform	Number of Subjects	Samples	Study Site
GSE120895	Heart Failure	GPL570	55 (47 HF + 8 Controls)	Endocardium tissue	Germany
GSE57338	Heart Failure	GPL11532	218 (82 HF + 136 Controls)	Left ventricle tissue	the United States
GSE31821	Atrial Fibrillation	GPL570	6 (4 AF + 2 Controls)	left atrial appendage	France
GSE41177	Atrial Fibrillation	GPL570	19 (16 AF + 3 Controls)	Left atrial appendage	Taiwan
GSE79768	Atrial Fibrillation	GPL570	13 (7 AF + 6 Controls)	Left atrial appendage	Taiwan

**Table 2:** GO terms in HF and AF common DEGs.

GO Term	Gene Ratio	Count	*p* Value	Q Value
GO-MF:0017147: Wnt-protein binding	0.40	2.00	2.43 × 10^−5^	3.33 × 10^−4^
GO-BP:0030856: regulation of epithelial cell differentiation	0.50	2.00	4.19 × 10^−4^	1.86 × 10^−2^
GO-BP:0090090: negative regulation of canonical Wnt signaling pathway	0.50	2.00	5.25 × 10^−4^	1.86 × 10^−2^

**Table 3:** KEGG pathways identified in heart failure DEGs.

KEGG Term	Gene Ratio	Count	*p* Value	Q Value
hsa04022: cGMP-PKG signaling pathway	0.19	3	4.22 × 10^−3^	1.44 × 10^−1^
hsa04976: Bile secretion	0.13	2	9.02 × 10^−3^	1.44 × 10^−1^
hsa01230: Biosynthesis of amino acids	0.13	2	9.76 × 10^−3^	1.44 × 10^−1^

**Table 4:** KEGG pathways identified in atrial fibrillation DEGs.

KEGG Term	Gene Ratio	Count	*p* Value	Q Value
hsa05140: Leishmaniasis	0.05	16	1.46 × 10^−8^	3.54 × 10^−6^
hsa05150: Staphylococcus aureus infection	0.05	15	2.43 × 10^−6^	2.94 × 10^−4^
hsa04145: Phagosome	0.06	18	1.41 × 10^−5^	1.14 × 10^−3^
hsa00190: Oxidative phosphorylation	0.05	16	3.49 × 10^−5^	2.11 × 10^−3^
hsa04672: Intestinal immune network for IgA production	0.03	9	7.15 × 10^−5^	3.14 × 10^−3^
hsa05012: Parkinson disease	0.05	16	7.79 × 10^−5^	3.14 × 10^−3^
hsa05016: Huntington disease	0.06	18	3.23 × 10^−4^	1.05 × 10^−2^
hsa05416: Viral myocarditis	0.03	9	3.58 × 10^−4^	1.05 × 10^−2^
hsa04514: Cell adhesion molecules (CAMs)	0.05	15	3.91 × 10^−4^	1.05 × 10^−2^
hsa05010: Alzheimer disease	0.05	16	6.66 × 10^−4^	1.49 × 10^−2^

## Data Availability

All data analyzed in this study are previously published and available at the NCBI (https://www.ncbi.nlm.nih.gov/geo/ (accessed on 20 March 2022)).

## Supplementary Materials

The following supporting information can be downloaded at: https://www.mdpi.com/article/10.3390/medicina58101319/s1, Figure S1: Heatmap of top 50 gene of GSE120895; Figure S2: Heatmap of top 50 gene of GSE57338; Figure S3: Heatmap of top 50 gene of atrial fibrillation datasets.

## References

[1] Tsao C.W., Aday A.W., Almarzooq Z.I., Alonso A., Beaton A.Z., Bittencourt M.S., Boehme A.K., Buxton A.E., Carson A.P., Commodore-Mensah Y. (2022). Heart Disease and Stroke Statistics-2022 Update: A Report from the American Heart Association. Circulation.

[2] Murphy S.P., Ibrahim N.E., Januzzi J.L. (2020). Heart Failure with Reduced Ejection Fraction: A Review. JAMA.

[3] Dries D., Exner D., Gersh B., Domanski M., Waclawiw M., Stevenson L. (1998). Atrial Fibrillation Is Associated with an Increased Risk for Mortality and Heart Failure Progression in Patients with Asymptomatic and Symptomatic Left Ventricular Systolic Dysfunction: A Retrospective Analysis of the Solvd Trials. Studies of Left Ventricular Dysfunction. J. Am. Coll. Cardiol..

[4] Bourassa M.G., Gurné O., Bangdiwala S.I., Ghali J.K., Young J.B., Rousseau M., Johnstone D.E., Yusuf S. (1993). Natural History and Patterns of Current Practice in Heart Failure. The Studies of Left Ventricular Dysfunction (Solvd) Investigators. J. Am. Coll. Cardiol..

[5] Middlekauff R.H., Stevenson W.G., Stevenson L.W. (1991). Prognostic Significance of Atrial Fibrillation in Advanced Heart Failure. A Study of 390 Patients. Circulation.

[6] Doval H.C., Nul D.R., Grancelli H.O., Perrone S.V., Bortman G.R., Curiel R. (1994). Randomised Trial of Low-Dose Amiodarone in Severe Congestive Heart Failure. Grupo De Estudio De La Sobrevida En La Insuficiencia Cardiaca En Argentina (Gesica). Lancet.

[7] Hindricks G., Potpara T., Dagres N., Arbelo E., Bax J.J., Blomström-Lundqvist C., Boriani G., Castella M., Dan G., Dilaveris P.E. (2021). 2020 Esc Guidelines for the Diagnosis and Management of Atrial Fibrillation Developed in Collaboration with the European Association for Cardio-Thoracic Surgery (Eacts): The Task Force for the Diagnosis and Management of Atrial Fibrillation of the European Society of Cardiology (Esc) Developed with the Special Contribution of the European Heart Rhythm Association (Ehra) of the Esc. Eur. Heart J..

[8] John R.M., Michaud G.F., Stevenson W.G. (2018). Atrial Fibrillation Hospitalization, Mortality, and Therapy. Eur. Heart J..

[9] Odutayo A., Wong C.X., Williams R., Hunn B., Emdin C.A. (2016). Prognostic Importance of Atrial Fibrillation Timing and Pattern in Adults With Congestive Heart Failure: A Systematic Review and Meta-Analysis. J. Card. Fail..

[10] Karnik A.A., Gopal D.M., Ko D., Benjamin E.J., Helm R.H. (2019). Epidemiology of Atrial Fibrillation and Heart Failure: A Growing and Important Problem. Cardiol. Clin..

[11] Wang T.J., Larson M.G., Levy D., Vasan R.S., Leip E.P., Wolf P.A., D’Agostino R.B., Murabito J.M., Kannel W.B., Benjamin E.J. (2003). Temporal Relations of Atrial Fibrillation and Congestive Heart Failure and Their Joint Influence on Mortality: The Framingham Heart Study. Circulation.

[12] Marrouche N.F., Brachmann J., Andresen D., Siebels J., Boersma L., Jordaens L., Merkely B., Pokushalov E., Sanders P., Proff J. (2018). Catheter Ablation for Atrial Fibrillation with Heart Failure. N. Engl. J. Med..

[13] Flaker G.C., Blackshear J.L., McBride R., Kronmal R.A., Halperin J.L., Hart R.G. (1992). Antiarrhythmic Drug Therapy and Cardiac Mortality in Atrial Fibrillation. The Stroke Prevention in Atrial Fibrillation Investigators. J. Am. Coll. Cardiol..

[14] Barrett T., Wilhite S.E., Ledoux P., Evangelista C., Kim I.F., Tomashevsky M., Marshall K.A., Phillippy K.H., Sherman P.M., Holko M. (2012). NCBI GEO: Archive for functional genomics data sets—Update. Nucleic Acids Res..

[15] Gautier L., Cope L., Bolstad B.M., Irizarry R.A. (2004). Affy—Analysis of Affymetrix Genechip Data at the Probe Level. Bioinformatics.

[16] Leek J.T., Johnson W.E., Parker H.S., Jaffe A.E., Storey J.D. (2012). The Sva Package for Removing Batch Effects and Other Unwanted Variation in High-Throughput Experiments. Bioinformatics.

[17] Koniari I., Artopoulou E., Velissaris D., Ainslie M., Mplani V., Karavasili G., Kounis N., Tsigkas G. (2021). Biomarkers in the Clinical Management of Patients with Atrial Fibrillation and Heart Failure. J. Geriatr. Cardiol. JGC.

[18] Kemp C.D., Conte J.V. (2012). The Pathophysiology of Heart Failure. Cardiovasc. Pathol..

[19] Corradi D. (2014). Atrial Fibrillation from the Pathologist’s Perspective. Cardiovasc. Pathol..

[20] Leyns L., Bouwmeester T., Kim S.-H., Piccolo S., De Robertis E.M. (1997). Frzb-1 Is a Secreted Antagonist of Wnt Signaling Expressed in the Spemann Organizer. Cell.

[21] Dann C.E., Hsieh J.-C., Rattner A., Sharma D., Nathans J., Leahy D.J. (2001). Insights into Wnt binding and signalling from the structures of two Frizzled cysteine-rich domains. Nature.

[22] Foulquier S., Daskalopoulos E.P., Lluri G., Hermans K.C.M., Deb A., Blankesteijn W.M. (2017). WNT Signaling in Cardiac and Vascular Disease. Pharmacol. Rev..

[23] Askevold E.T., Aukrust P., Nymo S.H., Lunde I.G., Kaasbøll O.J., Aakhus S., Florholmen G., Ohm I.K., Strand M.E., Attramadal A. (2014). The Cardiokine Secreted Frizzled-Related Protein 3, a Modulator of Wnt Signalling, in Clinical and Experimental Heart Failure. J. Intern. Med..

[24] Ren H., Luo J., Ouyang F., Cheng L., Chen X., Zhou H., Huang W., Zhang W. (2021). Polymorphism Is Associated with an Increased Risk of Essential Hypertension and Related Cardiovascular Diseases. Front. Cardiovasc. Med..

[25] Matsushima K., Suyama T., Takenaka C., Nishishita N., Ikeda K., Ikada Y., Sawa Y., Jakt L.M., Mori H., Kawamata S. (2010). Secreted Frizzled Related Protein 4 Reduces Fibrosis Scar Size and Ameliorates Cardiac Function after Ischemic Injury. Tissue Eng. Part A.

[26] Zeng W., Cao Y., Jiang W., Kang G., Huang J., Xie S. (2019). Knockdown of Sfrp4 attenuates apoptosis to protect against myocardial ischemia/reperfusion injury. J. Pharmacol. Sci..

[27] Ji Q., Zhang J., Du Y., Zhu E., Wang Z., Que B., Miao H., Shi S., Qin X., Zhao Y. (2017). Human epicardial adipose tissue-derived and circulating secreted frizzled-related protein 4 (SFRP4) levels are increased in patients with coronary artery disease. Cardiovasc. Diabetol..

[28] Wolke C., Antileo E., Lendeckel U. (2021). Wnt Signaling in Atrial Fibrillation. Exp. Biol. Med..

[29] Lin R., Wu S., Zhu D., Qin M., Liu X. (2020). Osteopontin induces atrial fibrosis by activating Akt/GSK-3β/β-catenin pathway and suppressing autophagy. Life Sci..

[30] Xiong Y., Wen S., Li Y., Wei Y., Fang B., Li C., Huang Q., Lin X. (2022). Comprehensive Analysis of Transcriptomics and Metabolomics to Illustrate the Underlying Mechanism of Helenalin against Hepatic Fibrosis. Eur. J. Pharmacol..

[31] Leventoux N., Augustus M., Azar S., Riquier S., Villemin J.P., Guelfi S., Falha L., Bauchet L., Gozé C., Ritchie W. (2020). Transformation Foci in Idh1-Mutated Gliomas Show Stat3 Phosphorylation and Downregulate the Metabolic Enzyme Etnppl, a Negative Regulator of Glioma Growth. Sci. Rep..

[32] Wang Y.Q., Chen W.C., Li K., Wu G., Zhang W., Ma P.Z., Feng S.Q. (2021). Tissue-based metabolomics reveals metabolic signatures and major metabolic pathways of gastric cancer with help of transcriptomic data from TCGA. Biosci. Rep..

[33] Tada Y., Yang P.C. (2017). Myocardial Edema on T2-Weighted Mri: New Marker of Ischemia Reperfusion Injury and Adverse Myocardial Remodeling. Circ. Res..

[34] Ivanova V., Sotnikov A., Melnikov M., Karpov A. (2020). Ultrastructural changes in the endocardium and endocrine cardiomyocytes in the wall of the left atrial appendage in patients with atrial fibrillation. Arkhiv Patol..

[35] Tan C., Zeng J., Wu G., Zheng L., Huang M., Huang X. (2021). Xinshuitong Capsule extract attenuates doxorubicin-induced myocardial edema via regulation of cardiac aquaporins in the chronic heart failure rats. Biomed. Pharmacother..

[36] Zhang J., Huang X., Wang X., Gao Y., Liu L., Li Z., Chen X., Zeng J., Ye Z., Li G. (2020). Identification of potential crucial genes in atrial fibrillation: A bioinformatic analysis. BMC Med. Genom..

[37] Harrison S.C., Zabaneh D., Asselbergs F.W., Drenos F., Jones G.T., Shah S., Gertow K., Sennblad B., Strawbridge R.J., Gigante B. (2013). A gene-centric study of common carotid artery remodelling. Atherosclerosis.

