# Supplementary material for: Screening and Bioinformatics Analysis of Crucial Gene of Heart Failure and Atrial Fibrillation Based on GEO Database
Source: Medicina (Kaunas). 2022 Sep 21;58(10):1319. doi: 10.3390/medicina58101319 (PMC9608246; doi:10.3390/medicina58101319)
Supplement: Supplementary file 1 [file medicina-58-01319-s001.zip › medicina-1848034-supplementary.pdf]

# Screening and Bioinformatics Analysis of Crucial Gene of Heart Failure and Atrial Fibrillation Based on GEO Database

**Author:**

Yuansong Zhuang[1], Zhentao Qiao[2], Xuanye Bi[1], Dongjian Han[1], Qingjiao Jiang[1], Yi Zhang[1], Fuhang Wang[1], Miaomiao Liu[1], Quanxu An[1], Jiahong Shangguan[1], Deliang Shen[1]

(1) Cardiology Department, First Affiliated Hospital of Zhengzhou University, Henan, China.

(2) Department of Vascular and Endovascular Surgery, First Affiliated Hospital of Zhengzhou University, Henan, China.

**Corresponding author:**

**Deliang Shen,** Cardiology Department, First Affiliated Hospital of Zhengzhou University, Henan, China. Tel:+86-15038335158. E-mail: dlshen@zzu.edu.cn

**Jiahong Shangguan,** Cardiology Department, First Affiliated Hospital of Zhengzhou University, Henan, China. Tel:+86-13613820323. E-mail: fccshangguangjh@zzu.edu.cn

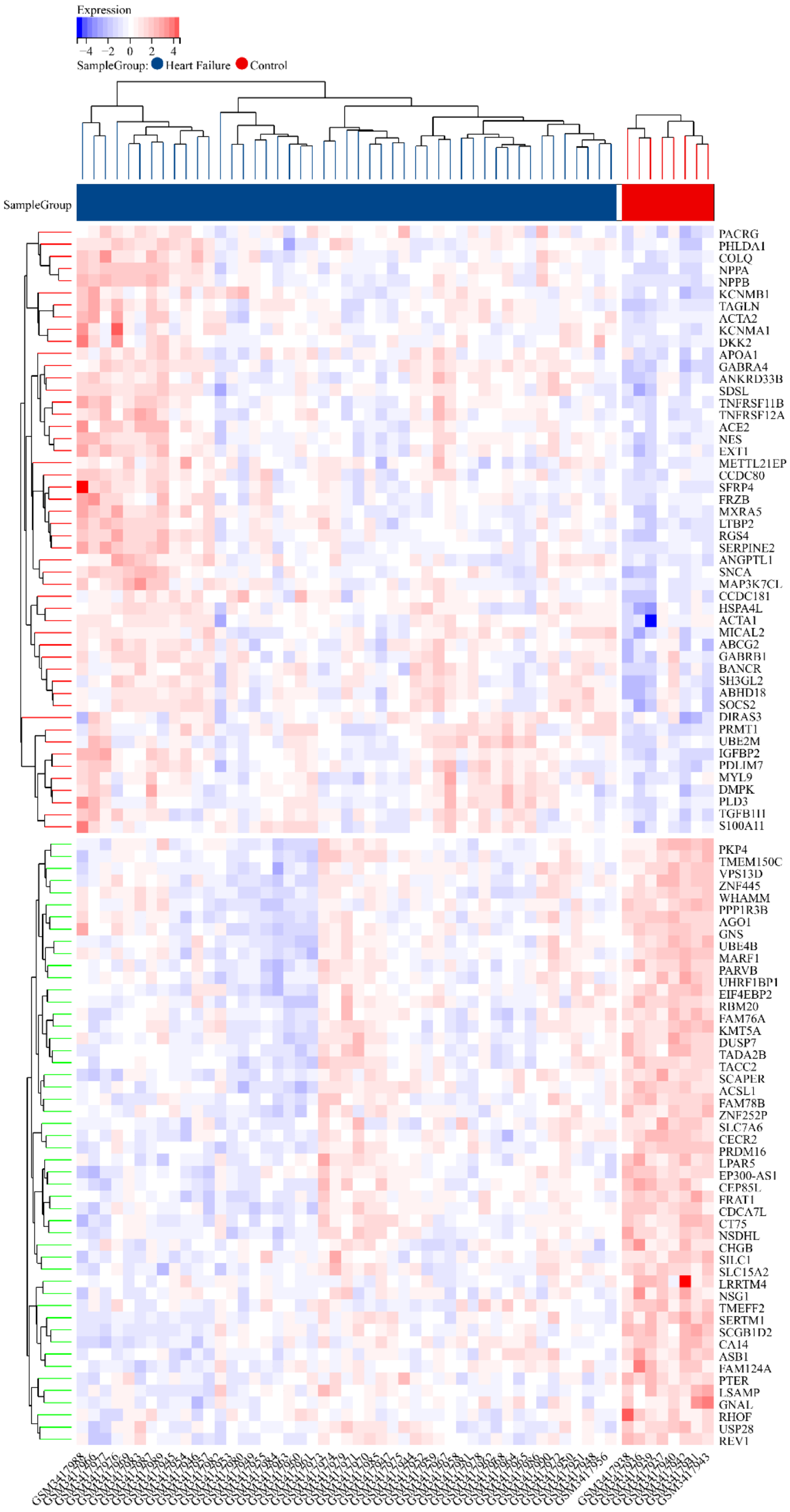


Figure S1 ： Heatmap of top 50 gene of GSE120895. In sample group column, blue represents the heart failure patient, and red represents the healthy person. The small cells in the picture are red for high expression gene and blue for low expression gene. This figure shows the top 50 up-regulated and down-regulated genes in GSE120895

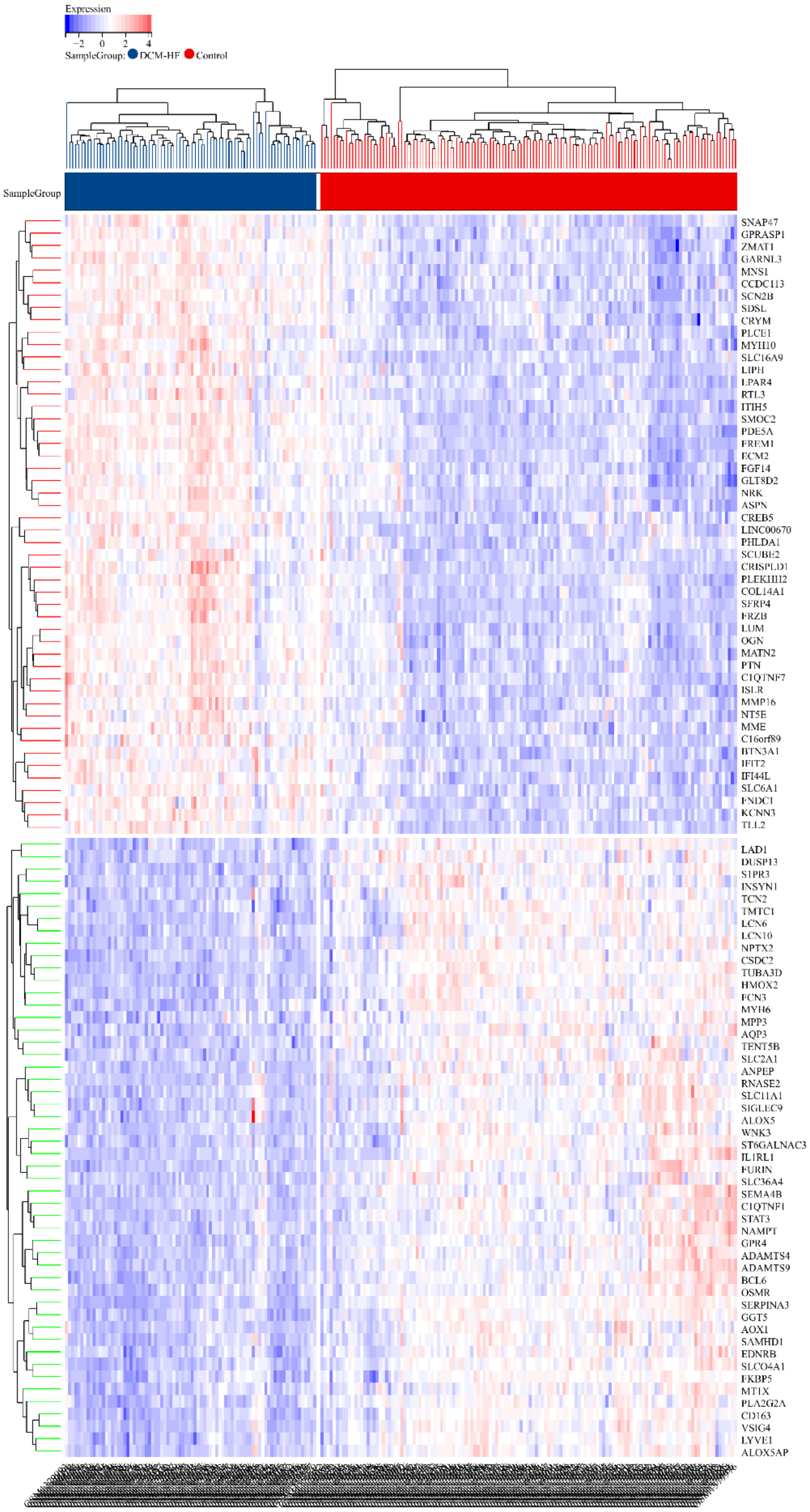


Figure S2 Heatmap of top 50 gene of GSE57338. In sample group column, blue represents the heart failure patient, and red represents the healthy person. The small cells in the picture are red for high expression gene and blue for low expression gene. This figure shows the top 50 up-regulated and down-regulated genes in GSE57338.

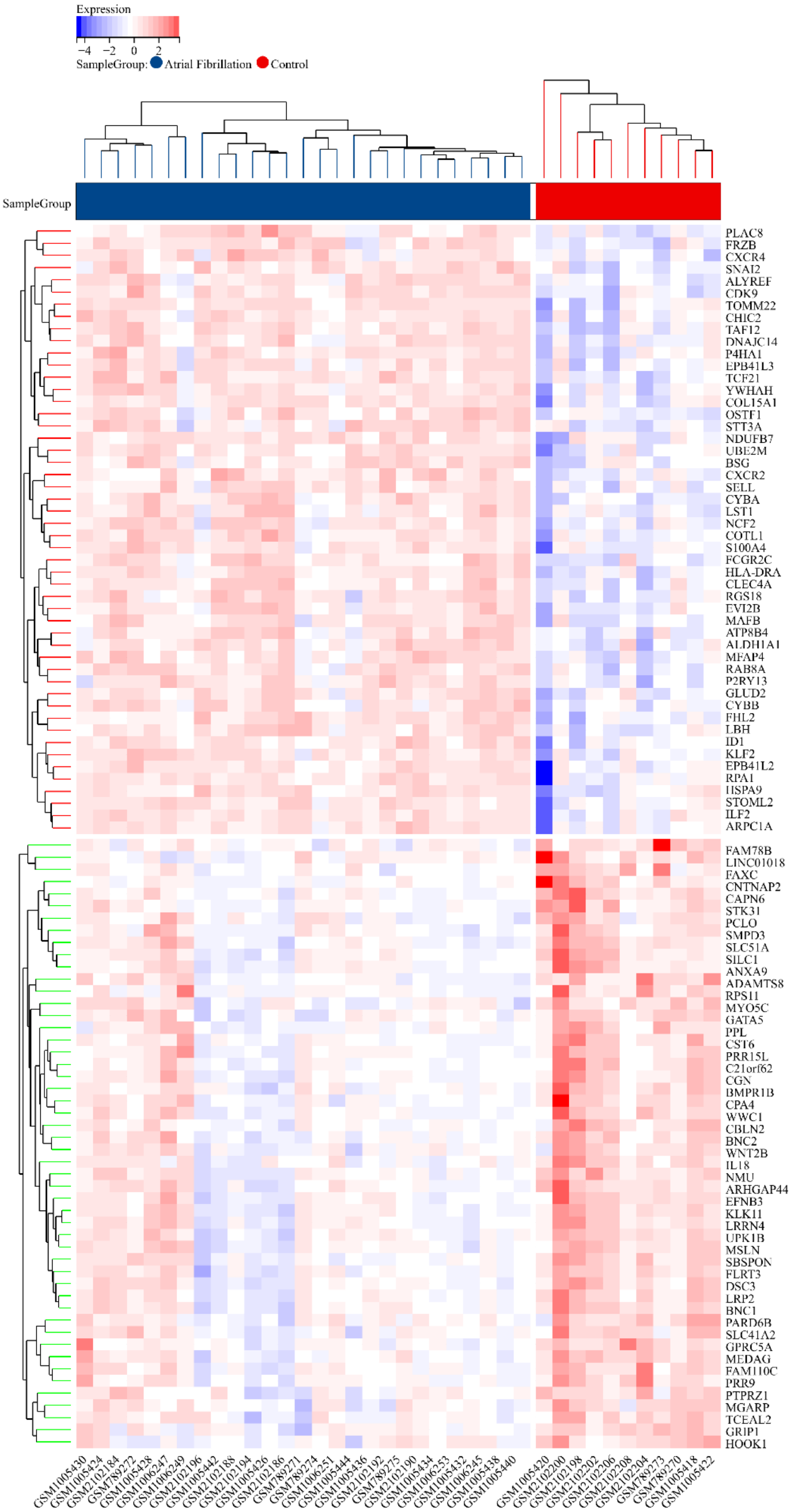


Figure S3 Heatmap of top 50 gene of atrial fibrillation datasets. In sample group column, blue

represents the atrial fibrillation patient, and red represents the healthy person. The small cells in the picture are red for high expression gene and blue for low expression gene. This figure shows the top 50 up-regulated and down-regulated genes in atrial fibrillation datasets.
